# The adherence to and utility of the Global Initiative for Chronic Obstructive Lung Disease guidelines for treating COPD among pulmonary specialists: a retrospective analysis

**DOI:** 10.1186/s12890-023-02503-7

**Published:** 2023-06-19

**Authors:** Fortune O. Alabi, Hadaya A. Alkhateeb, Mukudzeishe T. Zibanayi, Jica Garces, Kayla M. DeBarros, Pierina S. Benel Barletti, Kayla Garcia, Randall K. James

**Affiliations:** 1 Florida Lung Asthma and Sleep Specialists, Kissimmee, FL USA; 2 Philadelphia College of Osteopathic Medicine, Suwanee, GA USA; 3 Ross University School of Medicine, Bridgetown, Barbados

**Keywords:** COPD assessment test, Global Initiative for Chronic Obstructive Lung Disease, COPD classification, ICS, Triple therapy, Guideline adherence, CAT score

## Abstract

**Background:**

Despite the evidence-based guidelines promoted by the Global Initiative for Chronic Obstructive Lung Disease (GOLD), the overuse of prescription drugs to manage COPD, particularly inhaled corticosteroids (ICS), remains a persistent challenge. In this real-world study, we evaluated how patients with COPD were divided into ABCD groups based on the 2017 GOLD guidelines, determined the rate of adherence to the GOLD treatment recommendations, described the rate of ICS usage, and determined the rate of triple therapy (TT) prescription.

**Methods:**

The charts of 2291 patients diagnosed with COPD were retrospectively analyzed, of which 1438 matched the eligibility criteria.

**Results:**

The average patient age was 69.6 ± 10.9 years; 52% of patients were female. The average COPD assessment test (CAT) score was 18.3 ± 9.1. The ABCD breakdown was as follows: group A 19.5%, group B 64.1%, group C 1.8%, and group D 14.6%. All groups, except group D, showed discordance in COPD treatment relative to the proposed GOLD guidelines. Only 18.9% of group A and 26% of group B were treated in concordance with the guidelines. TT was primarily used in group D (63.3%) and overused in groups A (30.6%) and B (47.8%). ICS was overused in all groups, particularly in groups A (56.2%) and B (67.3%).

**Conclusion:**

Studies from the last decade have consistently revealed a lack of conformity between what physicians prescribe and what GOLD guidelines recommend. The excessive usage of ICS, which continues despite all the associated adverse effects and the attributable costs, is concerning. The awareness of GOLD guidelines among primary care physicians (PCPs) and respiratory specialists needs to be improved.

## Introduction

Chronic obstructive pulmonary disease (COPD) is a slowly progressive, inflammatory disease that causes airflow restriction and an irreversible loss of lung function. COPD is becoming increasingly common worldwide due to the rapid aging of the population. Approximately 16 million cases of COPD have been reported in the United States, and COPD is the third leading cause of death in the country [1]. The economic impact of COPD is substantial. The annual per-patient direct medical and hospitalization costs have been reported to be $10,367 and $6852, respectively. A study conducted in 2010 reported that prescription drug costs totaled $472 billion, whereas the total annual US payment for COPD care was $6.6 billion [2].

Despite evidence-based guidelines published and promoted by the Global Initiative for Obstructive Lung Disease (GOLD) science committee, the overuse of prescription drugs in COPD management, particularly the overuse of inhaled corticosteroids (ICS) at all stages of COPD, remains a persistent challenge. The impact of excessive medication usage on the rising costs of COPD management cannot be overlooked. Although smoking cessation and reducing COPD exacerbations significantly limit the increasing costs, the appropriate use of inhalers in this population would also reduce expenses. Primary care physicians (PCPs) in many countries have been accused of overprescribing ICS [3–5]. Moreover, this prescription pattern is not restricted to PCPs only. Safka et al. conducted their research in a respiratory department of an academic center in Canada. They discovered that 26.8% of the patients in GOLD group A received triple therapy (TT) and that 42.6% in GOLD group B likewise received TT [6].

The aim of this study was to describe the distribution of COPD in a nonacademic pulmonary specialty practice and to determine the adherence to the COPD GOLD 2017 recommendations.

### Objectives

The objectives of this study were to (1) determine how patients with COPD are divided into ABCD groups using the 2017 GOLD guidelines and (2) assess adherence to the 2017 GOLD recommendations.

## Methods

A retrospective analysis of all patients diagnosed with COPD, ICD-10 code J44.9, was performed at Florida Lung, Asthma & Sleep Specialists between January 2018 and December 2020. The study location is in Central Florida of the United State of America. A total of 2291 subjects were identified with the ICD-10 code J44.9 during that period. A minimum of 12 months of follow-up was required for inclusion in the study; thus, patients diagnosed with COPD after January 2020 were excluded from the analysis.

### Study participants

The eligible patients were men and women aged ≥ 40 years with physician-diagnosed COPD who needed therapy for ≥ 12 months. The inclusion criteria were as follows:

• The participants had a pulmonary specialist diagnosis of COPD with ICD code J44.9 for at least a year.
• The COPD assessment test was documented in patient charts.
• A history of exacerbation or no exacerbation was reported in patient charts.
• A history of exacerbation was defined as either hospitalization for COPD or outpatient treatment with steroids only or steroids and antibiotics.
• The participants received treatment for COPD at least on a rescue inhaler.
• Smoking history was documented in patient charts.
• Never smokers had significant secondhand exposure to tobacco or exposure to an environmental noxious substance or biomass fuel.
• The participants had a valid pulmonary function test (PFT) at the time of diagnosis.

### Exclusion criteria

The exclusion criteria were as follows:

• Patients with a concomitant diagnosis of asthma
• Patients with reversible airflow limitation as evidence by a post-bronchodilator response ≥ 12%
• Patients without COPD assessment test (CAT) scores in medical charts
• Participants without documented exacerbation history

The subjects included met all the eligibility criteria, which comprised both the inclusion and exclusion criteria.

### ABCD classification

The subjects that met eligibility criteria were classified into ABCD groups based on the 2017 GOLD guidelines. The ABCD classification system consists of two components: the assessment of symptoms using the modified Medical Research Council (mMRC) dyspnea scale or the COPD Assessment Test (CAT), and the assessment of exacerbation history in the previous year.

### Assessment of exacerbations

According to the GOLD guidelines, COPD exacerbation was defined as an acute event characterized by the worsening of a patient’s respiratory symptoms beyond normal day-to-day variations requiring a change in medication [7]. Exacerbation history was extracted from patient charts and entered as being present if the patient reported any of the following:

• Prescriptions for steroids or steroids and antibiotics, whether issued by the practice or by the patient’s other providers for a respiratory symptom.
• Admission to the hospital and urgent care or emergency room visits since the last office visit for respiratory symptoms were recorded as exacerbation episodes if steroids or steroids and antibiotics were prescribed.
• Any of these events occurring within the last 12 months.

### Assessments of symptoms

Symptom burden was assessed via the self-administered CAT. The CAT is an eight-item unidimensional measure of health status impairment in COPD, which yields a score between 0 and 40, with higher scores indicating a higher symptom burden [8].

Based on the combination of symptom assessment and exacerbation history, patients were classified into four groups: A, B, C, or D. Group A includes patients with low symptom burden (CAT < 10) and low risk of exacerbations (≤ 1 exacerbation without hospitalization in the last 12 months). Group B includes patients with high symptom burden (CAT ≥ 10) and low risk of exacerbations (≤ 1 exacerbation without hospitalization in the last 12 months). Group C includes patients with low symptom burden (CAT < 10) and high risk of exacerbations (≥ 2 exacerbations or ≥ 1 hospitalization in the last 12 months). Group D includes patients with high symptom burden (CAT ≥ 10) and high risk of exacerbations (≥ 2 exacerbations or ≥ 1 hospitalization in the last 12 months).

### Statistical analysis

The data were analyzed using SPSS version 24 software (IBM SPSS) and formulated into figures and tables. The data were expressed as the mean standard deviation if they were normally distributed or as the median (interquartile range) if they were nonparametric.

## Results

**Fig. 1:**
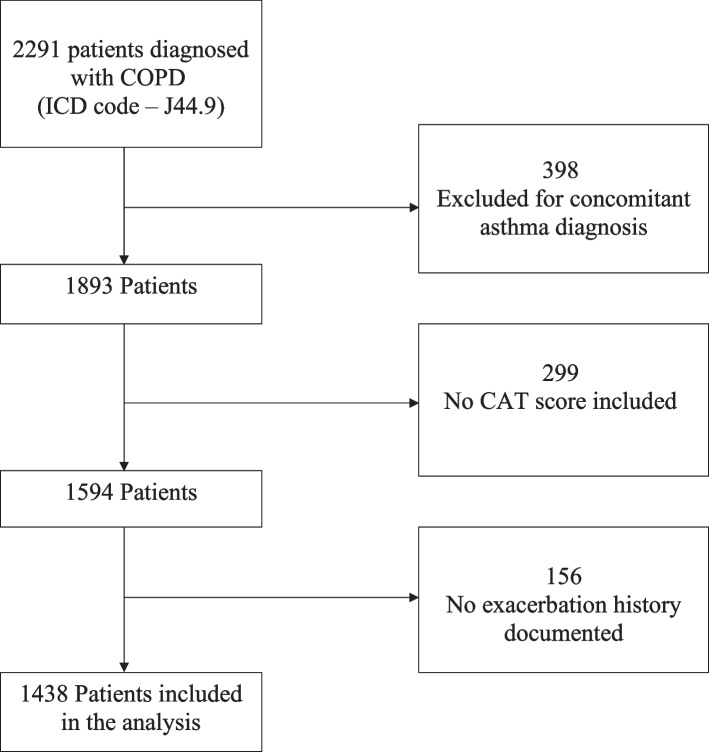
Inclusion and exclusion criteria. ICD = International Classification of Disease; CAT = COPD Assessment test

A total of 1438 of the 2291 patients screened between January 1, 2018, and December 31, 2020, met the inclusion criteria for this study (Fig. 1). In total, 398 patients were excluded because of concomitant asthma diagnosis, normal spirometry, or ≥ 12% bronchodilator response in FEV1 or FVC.

Table 1 presents the demographic data of the 1438 patients with COPD. Of these patients, 52% were female. The average age of the participants was 69.6 ± 10.9 years, and 84% of the population was White. Additionally, 18% of the patients were never smokers, and 82% were former or current smokers.

**Table 1:** Baseline characteristics of study population

Baseline Characteristics of Study Population					
**GOLD Category**	**A**	**B**	**C**	**D**	**TOTAL**
**Population**, n	281	921	26	210	1438
**Age, years (mean ± SD)**	69.7 (± 11.5)	69.6 (± 10.8)	69.3 (± 6.6)	69.6 (± 10.9)	69.6 (± 10.9)
**Male, n (%)**	140 (50)	447 (49)	19 (73)	79 (38)	685 (48)
**Female, n (%)**	141 (50)	474 (51)	7 (27)	131 (62)	753 (52)
**CAT Score, mean (± SD)**	7.8 (± 6.3)	20.5 (± 7.3)	7.1 (± 6.5)	24.4 (± 7.8)	18.3 (± 9.1)
**Race, n (%)**					
Asian	5 (2)	10 (1)	1 (4)	2 (1)	18 (1)
Black or African American	20 (7)	67 (7)	1 (4)	14 (7)	102 (7)
White	236 (84)	775 (84)	23 (88)	177 (84)	1211 (84)
Other Race	9 (3)	41 (4)	1 (4)	11 (5)	62 (4)
Declined to Specify	11 (4)	28 (3)	0 (0)	6 (3)	45 (3)
**Smoking history, n (%)**					
Current smoker	65 (23)	220 (24)	5 (19)	50 (24)	345 (24)
Former smoker	167 (59)	535 (58)	17 (65)	122 (58)	834 (58)
Never smoker	49 (17)	166 (18)	4 (15)	38 (18)	259 (18)

*CAT* COPD Assessment Test, *GOLD* Global Initiative for Chronic Obstructive lung Disease, *SD* Standard Deviation

Table 2 shows the spirometric measurements of the cohort. Fifty-one percent of the patients were in GOLD 2, whereas 20.6% and 6.26% were in GOLD 3 and 4, respectively. The average CAT score of the entire group was 18.3 ± 9.1.

**Table 2:** Spirometry and pharmacologic management of COPD

Spirometry and Pharmacologic Management of COPD				
	**Group A**	**Group B**	**Group C**	**Group D**
**Medications**				
**SABA**	18.86%	6.95%	3.85%	4.29%
**LAMA only**	0.00%	0.00%	3.85%	2.86%
**LABA only**	7.12%	6.73%	7.69%	3.33%
**ICS + LABA**	24.20%	17.92%	15.38%	15.71%
**LAMA + LABA**	17.79%	19.00%	34.62%	10.00%
**LAMA + LABA + ICS**	30.60%	47.77%	30.77%	63.33%
**ICS only**	1.42%	1.19%	3.85%	0.48%
**ICS + LAMA**	0.00%	0.43%	0.00%	0.00%
**Spirometry,** n (%)				
**Mild: FEV1 ≥ 80**	70 (25)	202 (22)	6 (23)	39 (19)
**Moderate: 50 ≥ FEV1 < 80**	145 (52)	470 (51)	13 (50)	106 (50)
**Severe: 30 ≥ FEV1 < 50**	51 (18)	193 (21)	5 (19)	47 (22)
**Very severe: < 30**	15 (5)	55 (6)	2 (8)	18 (9)

*SABA* short-acting bronchodilator, *LAMA* long-acting muscarinic antagonist, *LABA* long-acting beta agonist, *ICS* inhaled corticosteroid

**Fig. 2:**
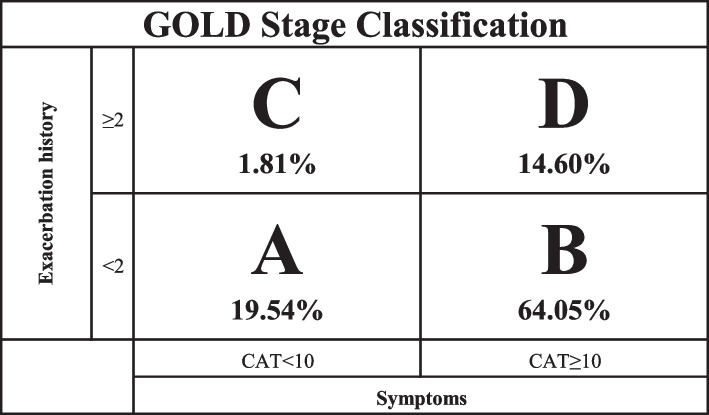
GOLD Stage classification of patients with COPD Seen at Florida Lung, Asthma & Sleep Specialists from 2018 – 2020. GOLD = Global Initiative for Chronic Obstructive Lung Disease; CAT = COPD Assessment Test

The ABCD categorization depicted in Fig. 2 demonstrates that group B was the largest group, comprising 64.1% of the study population. This was followed by group A, which accounted for 19.5% of the population. Group C only included 26 patients, representing 1.81% of the study population.

Table 2 shows the frequencies at which the different COPD inhalers were prescribed for each ABCD group of patients. Only 18.86% of the patients in group A were treated in concordance with the GOLD COPD 2017 guidelines. Most patients in group A received TT (closed and open devices). TT was prescribed to 30.6% of the patients in this group, with 71% of the TT being fixed, closed devices compared to 29.1% with nonfixed, open TT. In total, 26.0% of the patients in group B were treated in concordance with the COPD GOLD 2017 guidelines. A total of 17.9% received combination ICS and long-acting β_2_ agonist (LABA), and 47.8% were on TT, predominantly closed therapy in 67.5% of patients. Group C contained the smallest fraction of patients, accounting for 1.81% of the study population. The lowest percentage of discordance with the GOLD guidelines was found for group D. In total, 91.9% of the patients in this group were treated in concordance with preferred treatment guidelines. The use of TT was 63.3%; likewise, closed TT represented two-thirds of the TT usage in this group.

In total, 18.9% of the patients in group A were only receiving a short-acting β_2_ agonist (SABA) (Table 2). Monotherapy with long-acting muscarinic antagonist (LAMA) was not commonly used among the cohort of patients in this study. Monotherapy with LABA was used in approximately 7–8% of the patients in groups A, B, and C. The combination of LABA + LAMA was adopted primarily in group C, which comprised only a few patients compared to the other groups. The LABA + LAMA combination was equally used in 17.9% and 19.0% of groups A and B, respectively. TT (closed and open TT) was primarily used in group D at 63.3% and overused in groups A and B at 30.6% and 47.8%, respectively. The ICS + LABA combination was used to approximately the same extent in groups C and D at approximately 15.4% and 15.7%, respectively. The use of ICS + LABA was 24.2% in group A, which was higher than that in groups C or D and much higher than expected based on the GOLD 2017 preferred treatment guidelines (Table 2).

**Fig. 3:**
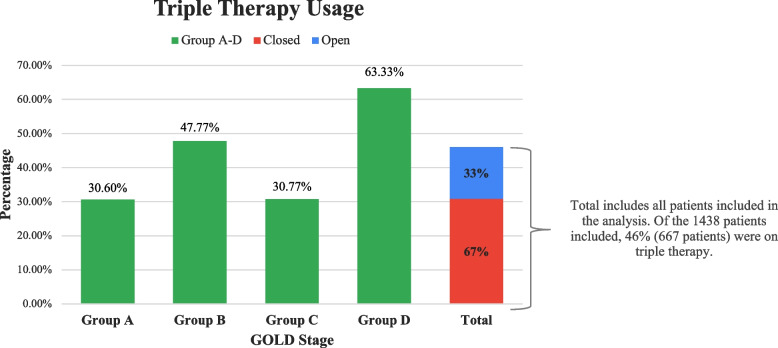
Triple therapy usage

ICS were overused in all groups (Table 3). ICS combined either with LABA only or as part of TT was used excessively in groups A and B. An ICS combination was used in 79.5% of patients in group D and 67.3% of patients in group B (Table 3). TT, via either closed or open devices, was used frequently in groups A and B, where it was used in 30.6% and 47.2% of patients, respectively. Overall, 46.4% of the patients in the study received TT, and 31% of the patients in the study received closed TT. Closed devices were mainly used among the patients on TT, accounting for 67% of all patients on TT compared to 33% on open TT devices (Fig. 3).

**Table 3:** Inhaled corticosteroid usage

Inhaled Corticosteroid Usage				
	**Group A**	**Group B**	**Group C**	**Group D**
**ICS + LABA + LAMA**	30.60%	47.77%	30.77%	63.33%
**ICS + LABA**	24.20%	17.92%	15.38%	15.71%
**ICS only**	1.42%	1.19%	3.85%	0.48%
**ICS + LAMA**	0.00%	0.43%	0.00%	0.00%
**Total**	56.23%	67.32%	50.00%	79.52%

*LAMA* long-acting muscarinic antagonist, *LABA* long-acting beta agonist, *ICS* inhaled corticosteroid

There was no association between FEV1 severity and adherence to the guidelines. FEV1 severity was evenly distributed across all four groups (Table 2). About 50% of patients in each group were in the moderate severity group (50 ≥ FEV1 < 80), making up most of the patient population. About 20% of patients were in the severe group (30 ≥ FEV1 < 50).

## Discussion

The combined COPD assessment proposed in the COPD GOLD guidelines 2017 separated the airflow limitation from ABCD grading, which is one of the significant revisions compared to the previous guidelines from 2011. The choice of treatment regimen should be based on the ABCD assessment tool, which considers the different phenotypes and intricacies of the disease process. Thus, patients with a predominantly symptomatic burden should receive long-acting bronchodilators, and those with an increased risk of exacerbation should receive inhalers that include ICS as one of the components.

The guidelines were modified to only reflect the symptom burden and exacerbation risk for classification [9]. Many retrospective studies have shown poor and inconsistent adherence to the treatment algorithm proposed by the GOLD guidelines [3, 10, 11]. This poor adherence is attributed to both PCPs and pulmonary specialists [6]. Many countries have reported significant disparities between the recommendations and real-world practice. The discordance between these GOLD guidelines remains a substantial challenge despite studies demonstrating the increased risk of exacerbation when there is misalignment between the GOLD guidelines and actual physician practice [12].

To the best of the authors’ knowledge, this is the first study to report the real-world management of COPD among pulmonary specialists and adherence to the 2017 GOLD guidelines. Safka et al. published their research at McMaster University in 2016. Their study, like this study, was retrospective and reflected the practice of pulmonologists from the Firestone Institute for Respiratory Health in Hamilton, Ontario. However, unlike this study, they compared the misalignment of COPD management to the 2011 GOLD guidelines [6]. The cohort in this study was collected from a single-specialty practice with ten pulmonary specialists and five nurse practitioners who were aware of the GOLD guidelines. The updated report was presented to the group in 2017. Davis et al.’s study from 2015 based on a survey of physicians in 12 countries regarding their knowledge and applications of the COPD management guidelines revealed that 58% of the PCPs reported awareness of the GOLD global strategy compared to 93% of the respiratory specialists [13].

This study’s results align with other published reports that addressed a similar subject. Not surprisingly, it was found that group C comprised the smallest fraction of patients, namely, 1.81% of the study population. Safka et al. showed that group C represented 4.2%. A different study in Poland reported 11.3% among specialists [14]. Unlike their study, groups A and D represented 8.2% and 59.2%, respectively. This study’s group A comprised 19.5% and group D 14.6% of the total cohort. This difference could be attributed to the referral bias because Safka et al.’s study was performed at a tertiary center where the patients’ severity might differ from a community-based practice. This referral bias might also account for the fact that group D contained 40.7% of participants in Wesolowski et al.’s study, which was performed among specialists, compared to 14.6% in this cohort but slightly comparable to Safka et al.’s study from Ontario, where group D was 59.2%. A community-based single-specialty pulmonary practice will have a mix of complex patients with frequent exacerbations and patients with garden variety COPD and mild COPD severity. It is also crucial to emphasize that both the Wesolowski and Safka studies were conducted before 2017 when the updated GOLD guidelines removed the effect of airflow limitation from the ABCD categorization. Another plausible explanation for the difference in the distribution of the COPD groups when compared to Safka et al.’s study could be that the CAT score was used for symptom assessment in this study. In the research from the United Kingdom by Price et al., group B was 67.2% when the CAT score was used for symptom assessment compared to 29.6% when the symptoms were assessed using the modified Medical Research Council (mMRC) score [3]. Similar to this study and others, group C comprised a small fraction of patients irrespective of the tool used to assess symptoms. In Price et al.’s study, group C comprised 8.4% according to the mMRC score and 2% by the CAT score. Group D in this study was much lower than the percentage reported in similar studies. In this study, 14.6% of the study population was in group D compared to 59.2% in Safka et al.’s study [6] and 40.7% in Wesolowski et al.’s study [14]. The assignment of patients into group D in these two studies was based on exacerbation history and evidence of severe airflow limitation. Contrary to this study, the placement into group D was strictly based on the exacerbation history. The exclusion of airflow limitation from the ABCD assessment tool could have contributed to the reduced percentage of groups C and D in our study because it is well known that the worse the airflow obstruction is, the higher the risk of exacerbations [15]. The retrospective nature of our study also presents the possibility of recall bias. Study participants may not accurately recall the number of exacerbations they have experienced since their last office visit. Underestimation of exacerbations would lead to a decreased placement into group D.

The COPD treatments for all the groups, except group D, were discordant relative to the proposed GOLD guidelines. In group D, 91.9% of the patients received GOLD guideline-recommended treatments. In total, 79% of the patients received an ICS-containing regimen either as an ICS + LABA combination or TT. LAMA was not commonly used alone in this study, but it was used in 73.3% of the patients in group D either as part of the TT or an LABA + LAMA combination. In Safka et al.’s study, LAMA was used in over 70% of the patients in group D, but whether it was used alone or in combination with LABA or as part of TT was not reported [6]. Only 18.9% of the patients in group A were treated in concordance with the GOLD treatment guidelines. The use of short-acting muscarinic antagonists (SAMAs) as rescue is not particularly common in practice. Hence, few patients received SAMAs; therefore, this variable was deliberately not abstracted during the data collection process in this study. TT was used in 30.6% of the patients in group A. However, in Safka et al.’s study, 26.8% of the patients in group A received TT. Bhatt et al. conducted a study of 21,711 patients with COPD on TT between 2014 and 2018. They found that 61.9% had exacerbation discordance, indicating that they were supposed to be in either group A or B [16]. In total, 74.3% of the patients in group B were treated discordantly with the GOLD treatment guidelines. LABA + LAMA was used in 19.0% of the patients. Nevertheless, the use of TT was substantial at 47.8% in group B. The increased percentage of patients belonging to GOLD groups A and B who were prescribed TT contrary to the GOLD recommendations is concerning. The overuse of TT in groups A and B has also been reported in other retrospective studies [17, 18]. The recommended treatment for group B is LABA or LAMA with escalation to the combination of both long-acting bronchodilators in those whose symptoms are very severe or who do not respond adequately to either one alone.

The use of TT in this group is expensive and associated with complications such as pneumonia [18]. An increased risk of nonfatal pneumonia is associated with 24 weeks of ICS use [19]. Disantostefano et al. reported a 20–50% increased risk of pneumonia in a new-user cohort study [20]. ICS usage has also been associated with an increased risk of cataract development [21, 22], hoarseness [23], fractures [24], skin bruising [25], progression of atypical mycobacterial infection [26], and increased risk of tuberculosis [27, 28]. TT was prescribed in 46.6% of the patients in group D, which is probably suitable in most cases. However, some of these patients were possibly maintenance naïve before starting TT. Maintenance naïve discordance is common among patients who are administered TT. Bhatt et al. reported that 34.4% of 21,711 patients who began TT from 2014 to 2018 were not receiving any maintenance inhaler before initiating TT [16]. The true percentage of patients with COPD requiring TT is not clearly known. However, in the Bhatt et al. study with a large cohort of patients, 25.9% of the patients were prescribed TT in alignment with the GOLD recommendations. Importantly, most patients on TT in real-world practice would not have met the inclusion criteria in most randomized control trials (RCTs) of TT. For example, in the DACCORD trial [29], which was a prospective, noninterventional, real-world study performed in Germany, most of the patients receiving TT would not have met the inclusion criteria used in the RCT. Only 1.8%, 5.4%, and 24% of the DACCORD cohort met the inclusion criteria of TRIBUTE [30], IMPACT [31], and KRONOS [32], respectively.

Another staggering finding in this study was the excessive usage of ICS in all the groups. According to the GOLD 2017 guidelines, ICS usage in combination with LABA or the LABA + LAMA combination is an acceptable treatment for patients who continue to exhibit exacerbations despite treatment with LAMA. It is also acceptable in patients with concomitant asthma or elevated sputum or blood eosinophils. In this study, ICS was used as a part of TT or combined with LABA in 56.2% and 67.3% of groups A and B, respectively. When both groups were combined, 778 patients received ICS, representing 54% of the study population. In Vestbo et al.’s study, a cross-sectional survey performed across European countries and the United States, 38.8% and 51.8% of patients in groups A and B, respectively, received ICS, which is contrary to the recommendations [33]. Similarly, an Italian study on the use of medications in more than one million patients revealed that 55.6% of the patients with COPD were on ICS without having exacerbation risk [34]. The overuse of ICS is not restricted to PCPs only. A cross-sectional observational study conducted among 49 pulmonary units across Italy by Corrado et al. demonstrated that ICS was used alone or in combination with LABA in 15.2% and 66.8%, respectively, of the patients with COPD enrolled in the study [35]. The overuse of ICS continues unabated despite numerous well-known adverse effects of ICS [36]. The increased risk of pneumonia is a well-known adverse effect, as shown in numerous studies [37, 38]. Likewise, ICS-containing devices have been found to have a numerical mortality advantage in a few RCT trials [31, 39]. Notably, ICS may be protective against death in patients with COPD admitted with pneumonia [40]. Several reasons are attributed to the excessive use of ICS-containing devices by both PCPs and respiratory specialists. They include, but are not limited to, the following observations: (A) There is a lack of awareness of current guidelines and recommendations, more so among PCPs than specialists. (B) There is uncertainty in the diagnosis, especially among PCPs who may not have the capacity to perform spirometry in their offices and occasionally among specialists when the PFT report is complicated by findings of significant post-bronchodilator FEV1 with abnormal diffusion capacity or when patients with a childhood history of asthma with significant reversibility but the presence of centrilobular emphysema on the CT scan of the chest from years of excessive smoking. In such cases, some providers are more likely to err and prescribe devices containing ICS rather than long-acting bronchodilators. Therefore, in essence, an uncertain diagnosis leads to misalignment between recommendations and real-life practice style. (C) Some respiratory specialists will prescribe ICS-containing devices to patients with major allergic components to their symptoms or those with elevated blood eosinophilia ≥ 2% or absolute blood eosinophils of ≥ 150 cells per microliter. (D) The providers are merely prescribing the inhalers that the third-party payers (insurance companies) will cover. There may be patients in group B who could have fared well on LABA or LAMA, but the carrier does not cover LABA or LAMA. Instead, the patients are prescribed the SABA and ICS + LABA combination in their formulary. Therefore, the conundrum is either to refrain from prescribing a maintenance inhaler or prescribe whatever the insurance companies are willing to cover, which may not align with the GOLD recommendations. (F)  The concomitant diagnosis of asthma is made in as many as 23% of COPD patients [41]. However, efforts have been made to exclude patients with asthma during data review and collection to limit their influence on the study analysis. (G) Some providers prescribe ICS to patients with COPD and eosinophilia. Eosinophilic airway inflammation has been proposed as a marker that may identify ICS responsiveness [42, 43]. (H) The use of TT, either via open or closed devices, has been increasing since 2018, when the first closed TT arrived on the market. Similar to this study and many others, closed TT accounts for most TTs. It is possible that closed TT is being prescribed more recently because it negates the need to differentiate asthma from COPD, and it may also reflect the simplification of therapy. (I) Some suggestions with some numerical advantages indicate that ICS might reduce the decline in FEV1 in COPD patients [44]. It is difficult to discover if this information might be the impetus for using ICS more than the recommended guidelines. Nevertheless, the effect of ICS on FEV1 decline is still questionable and remains to be proven in an RCT [45]. (J) The managed care companies and their primary care providers are sometimes reluctant to refer patients to a specialist, resulting in delayed diagnosis and, at times, wrong diagnosis and mismanagement. Some managed healthcare companies also have limited drugs for managing COPD on their formularies, limiting the ability of the provider to prescribe guideline-recommended treatments. The inappropriate use of maintenance inhalers and the excessive use of ICS are contributing factors to the sky-rocketing expenses of managing COPD despite a slight decrease in prevalence.

## Limitations

This study has many limitations because of its retrospective nature. The study utilized the 2017 COPD GOLD recommendations because that was the first update that separated airflow limitation from ABCD severity grading. The percentage of patients with no exacerbations was determined by reviewing patient charts and searching for the prescription history for antibiotics and steroids used since their last visit. The distinction between exacerbation + and exacerbation – is significantly affected by the patient’s ability to recollect their history. This deficiency was mitigated by assessing the pharmacy records during the chart review and data abstraction to determine whether they were prescribed steroids or antibiotics. Because the CAT score is only used in practice for symptom assessment, the GOLD group staging–based mMRC score could not be provided. It has been suggested that the CAT score compared to the mMRC score may overestimate group B patients. Nonpharmacological management, such as smoking cessation, adherence to oxygen, referral for pulmonary rehabilitation, and compliance with vaccinations, were not assessed in this study. These are all crucial aspects of providing comprehensive management to patients with COPD, and their omissions do not aim to lessen their significance. The use of Daliresp and azithromycin in group D patients to reduce exacerbation was also not assessed. Some of the patients included in the analysis with COPD diagnosis did not meet the spirometric criteria for COPD as defined by the GOLD guidelines. It was believed that including these patients would be more reflective of real-world patients with COPD that are seen in community-based practice. This study primarily aimed to address the misalignment between GOLD 2017-guided recommendations and real-world pulmonary practice in the usage of maintenance inhalers in patients with COPD. Patients with reversible airflow limitation as evidenced by a post-bronchodilator response ≥ 12% were deliberately excluded from the study as most patients with significant reversibility were preferentially given a diagnosis of asthma or asthma with COPD overlap syndrome (ACOS) and were almost always being prescribed ICS. They were excluded in order to prevent overestimation of ICS usage in this study.

## Conclusion

Observational studies of PCPs and specialists in the last decade have consistently revealed a lack of conformity between what physicians prescribe and what the GOLD strategy recommends. Most previous studies were conducted before the revised 2017 GOLD guidelines. Despite eliminating the airflow limitation for categorizing patients by the group ABCD scheme, the discordance between guideline recommendations and real-world practice continues. In particular, the excessive usage of ICS continues with all the associated adverse effects, and the attributable costs remain a significant challenge. The awareness of GOLD guidelines among PCPs needs to be improved. Notably, the reasons for poor adherence to the recommendations by respiratory specialists need to be better understood to provide corrective guidance. The continual education of all providers (PCP and respiratory specialists) regarding timely diagnosis, timely referral when the diagnosis is in doubt, recommended treatment pathways, and the rationale behind the guidelines is recommended. Notwithstanding, the authors are disturbed by the chasm between the guideline recommendations and real-world practice in COPD management. Understanding the reasons for this gulf between the GOLD guideline recommendations and real-world medical practice requires further research.

## Data Availability

The datasets used and/or analyzed during the current study are available from the corresponding author on reasonable request.

## Abbreviations

GOLD: Chronic Obstructive Lung Disease
ICS: Inhaled corticosteroid
TT: Triple therapy
CAT: COPD assessment test
PCP: Primary care physician
COPD: Chronic obstructive pulmonary disease
PFT: Pulmonary function test
SPSS: Statistical Package for the Social Sciences
FEV1: Forced expiratory volume in one second
FVC: Forced vital capacity
LABA: Long-acting β_2_ agonist
SABA: Short-acting β_2_ agonist
LAMA: Long-acting muscarinic antagonist
SAMA: Short-acting muscarinic antagonists
mMRC: Modified Medical Research Council
RCT: Randomized control trial
